# A Validated RP-HPLC Stability Method for the Estimation of Chlorthalidone and Its Process-Related Impurities in an API and Tablet Formulation

**DOI:** 10.1155/2020/3593805

**Published:** 2020-04-10

**Authors:** Chaitali Kharat, Vaishali A. Shirsat, Yogita M. Kodgule, Mandar Kodgule

**Affiliations:** ^1^Bombay College of Pharmacy, Kalina, Santacruz East, Mumbai 4000098, India; ^2^IQGEN-X Pharma Pvt. Ltd., A-165, Khairane Road, Sector 2, Kopar Khairane, Navi Mumbai 400710, India

## Abstract

Low-dose thiazide and thiazide-like diuretics are widely used as first-line therapy for hypertension. Chlorthalidone, a monosulfamyl diuretic, is frequently prescribed in cases of hypertension and congestive heart failure. In this research paper, an improved reverse-phase HPLC method was developed for the simultaneous identification and quantitation of pharmacopoeia-listed and in-house process- and degradation-related impurities of chlorthalidone in bulk drug and formulations. Chromatographic separation was carried out on a C_8_ column (250 × 4.6 mm; ‘5 *μ*m particle size) at a flow rate of 1.4 mL/min with a 220 nm detection wavelength. Mobile phase A consisted of buffer solution (diammonium hydrogen orthophosphate (10 mM, pH 5.5)) and methanol at a 65 : 35 ratio (v/v), and mobile phase B consisted of buffer solution and methanol at a 50 : 50 ratio (v/v). The API and formulation were subjected to stress conditions such as acid, alkali, oxidation, thermal, and photolytic conditions. Validation studies for the in-house process impurities were performed for specificity, limit of detection (LOD), limit of quantitation (LOQ), linearity, precision, accuracy, and robustness. Thus, an improved RP-HPLC method capable of good separation of all known and unknown impurities with acceptable resolution and tailing factor was developed.

## 1. Introduction

Pharmaceutical impurities are unwanted chemicals that remain with active pharmaceutical ingredients (APIs) or drug product formulations. Impurities in drug substances may be generated during synthesis or derived from starting materials, intermediates, reagents, solvents, catalysts, or reaction by-products. Impurities may also be formed during drug product development as a result of the inherent instability of drug substances, incompatibility with added excipients, or interactions with packaging materials. The amounts of various impurities found in drug substances will determine the ultimate safety of the final pharmaceutical product. Therefore, the identification, quantitation, qualification, and control of impurities are a very critical part of the drug development process.

Various regulatory authorities, such as the International Conference on Harmonization (ICH), the United States Food and Drug Administration (USFDA), and the European Medicines Agency (EMA), focus on the control of impurities. Additionally, a number of official compendia, such as the British Pharmacopoeia (BP), the United States Pharmacopeia (USP), the Japanese Pharmacopoeia (JP), and the European Pharmacopoeia (EP), have included specification limits that restrict impurity levels present in APIs as well as in drug formulations [1–4]. An impurity profile is a complete study of the identified and unidentified impurities present in a batch of API produced by a specific controlled production process that monitors the same impurities throughout formulation development. This helps to identify the risk associated with the toxicity of any drug when consumed by patients. Thus, numerous articles have been published that describe stability and indicate analytical methods for impurities and forced degradation products in pharmaceuticals [5–14].

**Figure 1:**
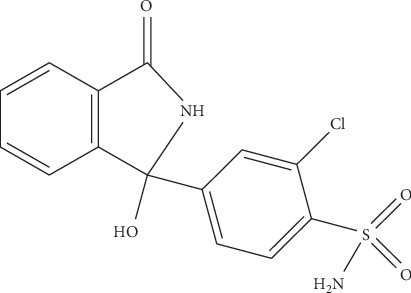
Structure of chlorthalidone.

Hypertension is one of the major causes of the increased mortality rate in the past decade and is increasing exponentially due to the current unhealthy lifestyles. Diuretics are frequently recommended as first-line therapy for hypertension. Chlorthalidone chemically as 2-chloro-5-(l-hydroxy-3-oxo-l-isoindo-linyl) benzenesulfonamide (Figure 1), is an oral diuretic administered alone and in combination with antihypertensive agents [15, 16].

Singer et al. reported that the intermediate products formed during chlorthalidone synthesis, such as 3-(4-chlorophenyl) isoindolin-1-one (stage II) and penultimate intermediate 3-(4-chlorophenyl)-5-sulfonamide isoindoline-1-one (stage III), are likely to be present in the final chlorthalidone bulk drug [17]. Different analytical methods for the determination of chlorthalidone impurities have been reported. The reported methods describe degradation studies and the estimation of assay and impurity profiles for both drug substance and drug product, as well as in combination with other drug substances [18–23]. The proposed analytical method can be performed effectively on chlorthalidone API and chlorthalidone tablets for known EP-specified, process-related, and degradation impurities. A study performed by Marineni and Sreenivasulu Reddy showed the separation of chlorthalidone, telmisartan, and its impurities, though only two chlorthalidone impurities (impurity A and impurity B) were separated with good resolution. The article does not mention the compound's specificity for degradation impurities [20]. A stability-indicating method was developed by Sonawane et al. for bulk drug and tablets using an experimental design [21]. An ultra-HPLC method was developed for fixed dose combinations of azilsartan, medoxomil, and chlorthalidone by Quaglia et al. [22]. The two specified impurities and chlorthalidone API were separated by Samanthula et al. by HPTLC [23].

**Figure 2:**
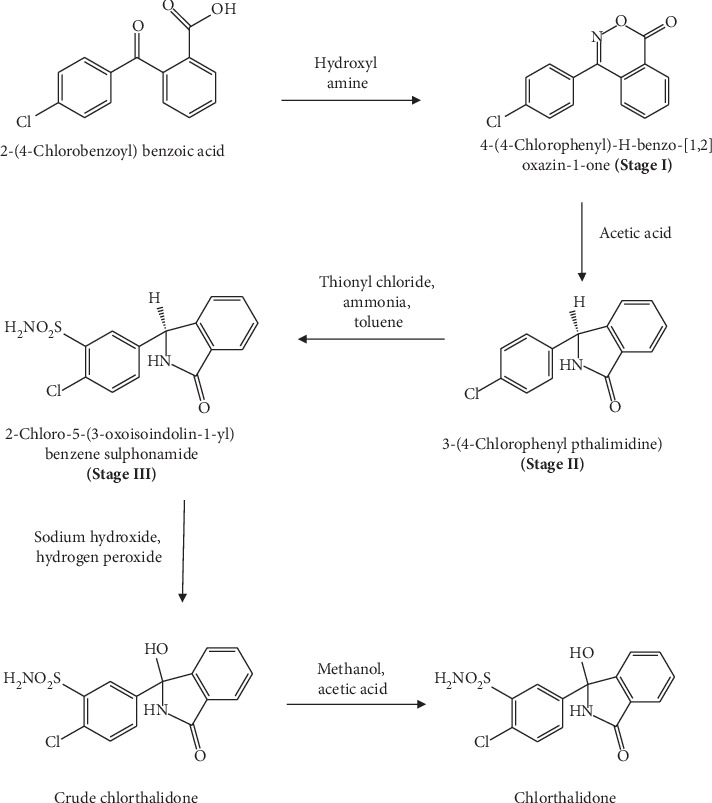
Synthesis scheme for chlorthalidone API.

An HPLC method for the identification and quantitation of chlorthalidone API-related impurities, described as pharmacopoeia-listed impurities, has been mentioned in the European Pharmacopoeia [24]. The synthesis of chlorthalidone API was performed by IQGEN-X Pharma Private Limited, Navi Mumbai, India (Figure 2). Initial evaluation of an EP method for HPLC analysis did not indicate resolution between chlorthalidone, pharmacopoeia-listed impurities, and in-house process-related impurities. Therefore, a new HPLC method had to be developed with modifications to the EP method to separate the EP-listed impurities, in-house process-related impurities, and degradation impurities of chlorthalidone API, as well as those from tablet formulation.

Thus, the objective of the current work was to check the quality of chlorthalidone API by detecting the potential impurities throughout drug product development, i.e., from the API stage to the formulation stage using an HPLC method to qualitatively and quantitatively analyse all the analytes in the API and formulation (i.e., EP-listed, process-related, and degradation impurities).

The EP-specified and in-house process-related impurities generated through the above synthetic pathway are shown in Table 1 and are detected throughout this work.

## 2. Experimental

### 2.1. Instrumentation

FT-IR spectra of the stage I, II, and III samples were recorded in a solid state, such as KBr dispersion, using a PerkinElmer FTIR spectrophotometer. ^1^H NMR spectra of the intermediate products were recorded on a Bruker 400 MHz NMR spectrometer in DMSO (Merck, India) as the solvent. Mass spectra of the stage I, II, and III samples were recorded on the AB SCIEX-API 4000™ system by dissolving the sample in H_2_O: methanol (50 : 50% v/v) and infused directly. The m/z of the molecular ion peak was determined (spectral data are added in supplementary data). The analytical method development and validation were performed on a high-performance liquid chromatograph (Waters Alliance e2695 Separations module with a PDA detector) equipped with a quaternary solvent delivery pump, degasser, autosampler, and column thermostat using the Empower Software Version 4 data handling system (Waters Corporation, Milford, MA, USA).

### 2.2. Material and Reagents

Chlorthalidone API (100.0%), chlorthalidone tablets, EP impurities, and in-house process impurities were obtained from the In-house R&D laboratory at IQGENX Pharma Private Limited (Kopar Khairane, Navi Mumbai, Maharashtra). Reagents such as hydrochloric acid (35% v/v) (EMPARTA grade), orthophosphoric acid (EMPARTA grade), sodium hydroxide (EMPLURA grade), diammonium hydrogen orthophosphate (Excela-R grade), and HPLC grade solvents (acetonitrile and methanol) were procured from MERCK, Mumbai, India. Hydrogen peroxide (30% v/v) was obtained from SD Fine Chemicals. Water from a Milli-Q water purification system was used for the HPLC analysis.

### 2.3. Chromatography Conditions

The mobile phase consisted of buffer solution (diammonium hydrogen orthophosphate (10 mM, pH 5.5)) and methanol at a 65 : 35 ratio (v/v) as mobile phase A and at a 50 : 50 ratio (v/v) as mobile phase B. Both mobile phases were filtered through a 0.45 *μ*m PVDF filter membrane and degassed under vacuum before use. The chromatographic column used was Zorbax RX C_8_ (dimensions 250 × 4.6 mm and particle size 5 *μ*m). The analytes were identified with the help of a PDA detector. The detection wavelength was set at *λ*_max_ of 220 nm. All separations were performed using the gradient mode at 40°C with a 1.4 mL/min flow rate, 20 *μ*L injection volume, and 60 min run time. The mobile phase elution programme used for standard, blank, placebo, and sample solution is specified in Table 2.

### 2.4. Analytical Procedure

#### 2.4.1. Preparation of Solutions

*Diluent*. Buffer solution, methanol, and sodium hydroxide solution (2 g/L) were mixed at a 50 : 48 : 2 ration (v/v/v), respectively. This diluent solution was used for the preparation of the solutions.

*Blank*. Diluent was used as a blank.

*Standard Solution*. 500 *μ*g/mL chlorthalidone standard solution was prepared and diluted to make a solution with a concentration of 20 *μ*g/mL. This solution was further diluted to give a solution with a final concentration of 2 *μ*g/mL and was used for system suitability studies.

The 100 *μ*g/mL standard solutions for the stages II and III process impurities and chlorthalidone were prepared individually using diluent. These solutions were further diluted to give individual working standard solutions with a concentration of 10 *μ*g/mL. These solutions were used for the validation studies.

*Sample Solution*. A 1000 *μ*g/mL in-house synthesized chlorthalidone API sample was prepared. The tablets were punched using the in-house chlorthalidone API, which was light green in colour and oval. The scored tablets were debossed with S to the left of the score and 9 to the right of the score on one side of the tablet. The average tablet weight was 140.5 mg. The average weight for 20 tablets was determined, and then they were crushed into a fine powder with a mortar and pestle. Tablet powder equivalent to 100 mg of chlorthalidone was weighed, and a 1000 *μ*g/mL chlorthalidone tablet solution was prepared. The placebo comprised a mixture of excipients, such as microcrystalline cellulose, sodium starch glycolate, pregelatinized starch, colloidal silicon dioxide, stearic acid, and lake blend green. The placebo solution was prepared without the use of chlorthalidone API.

### 2.5. Validation

#### 2.5.1. Specificity

Specificity is the ability of the method to measure analyte responses in the presence of potential impurities. The in-house process-related impurity working standard solution was spiked with chlorthalidone API and the tablet formulation at specific concentrations. The resolution, RT, RRT, purity angle, and purity threshold of chlorthalidone and in-house process-related impurities were determined.

Stress testing of the drug substance can help to identify possible degradation products, which can help to validate the stability-indicating power of the analytical procedure used. The force degradation studies were performed on API and tablet formulations under varying conditions such as acid hydrolysis, alkali hydrolysis, and oxidative hydrolysis. All the stress degradation studies were performed with an initial drug concentration of 1000 *μ*g/mL. Acid hydrolysis was performed in 1 N HCl at 60°C for 30 min. Base hydrolysis was carried out in 1 N NaOH at 60°C for 30 min. Oxidation studies were performed in 30% v/v H_2_O_2_ at 60°C for 30 min.

#### 2.5.2. Linearity

The standard solutions for linearity were prepared at 50% to 140% of the concentration of chlorthalidone and the stage II and III process impurities. Linearity solutions for stage II and III process impurities were prepared from working standard solutions at five different concentrations, i.e., 0.75, 1.05, 1.5, 1.8, and 2.1 *μ*g/mL. For the chlorthalidone linearity studies, solutions with concentrations of 1.0, 1.4, 2, 2.4, and 2.8 *μ*g/mL were used. Method linearity was evaluated by drawing a calibration curve and showing the plot of the impurity area versus the concentration. The regression equation, correlation coefficient (*r*^2^), slope, and intercept values of the calibration curves were determined. The linearity test solutions were injected in triplicate.

#### 2.5.3. LOD and LOQ

These studies were carried out using a residual standard deviation method and with a visual evaluation method. The LOD and LOQ were calculated using the following formulas: LOD = 3.3 × *σ*/*S* and LOQ = 10 × *σ*/S, where *σ* is the standard deviation of the response and *S* is the slope of the calibration curve of the respective analyte. The LOD and LOQ studies performed with the visual method were carried out by injecting diluted working standard solutions of the impurities and chlorthalidone. A precision study was carried out at LOQ with six injections (*n* = 6). The concentration and peak area for each individual impurity and chlorthalidone were calculated.

#### 2.5.4. Precision

The system precision study was carried out by injecting a 2 *μ*g/mL chlorthalidone standard solution six times, and the %RSD of the peak area was calculated. Similarly, for the method precision, working standard solutions of the impurities and chlorthalidone were mixed and prepared at 1.5 and 2 *μ*g/mL, respectively. The mixture of chlorthalidone and both process impurities was injected six times, and %RSD of the %w/w impurities was calculated.

#### 2.5.5. Accuracy

For the chlorthalidone API and tablet formulation accuracy study, target impurity concentrations were selected as per their specification limits. The concentration of chlorthalidone (1000 *μ*g/mL) in the API and formulation was constant throughout the accuracy study. The accuracy of the method was examined by spiking working standard solutions of stage II and III process-related impurities from the API and formulation. Spiking was achieved by adding 1.5 mL, 3 mL, and 4.5 mL corresponding to impurity concentrations of 0.75, 1.5, and 2.25 *μ*g/mL, respectively, at 50, 100, and 150% concentration levels. The percent recovery, standard deviation (SD), and %RSD were calculated for both in-house process impurities.

#### 2.5.6. Robustness

The robustness of the method was determined by varying the original buffer pH of 5.5 by ±0.2, the mobile phase flow rate of 1.4 mL/min by ±0.2 mL/min, and the wavelength at 220 nm by ±2 nm. Evaluation of the results was carried out by determining any change in the relative retention time (RRT) and peak areas for chlorthalidone and the in-house process impurities.

#### 2.5.7. Solution Stability

Benchtop stability was performed to ensure that the in-house process impurities and chlorthalidone working standards remained stable in the diluent (buffer : methanol : sodium hydroxide at a ratio of 50 : 48 : 2). These solutions were stored at room temperature and analysed at 0, 18, 36 and 40 hr time intervals.

## 3. Results and Discussion

According to the developed synthesis process, the two generated in-house process impurities, intermediate stage II and intermediate stage III, were specific to this chlorthalidone synthesis process. Thus, developing an analytical method that can detect known EP-reported impurities, in-house process impurities, and degradation impurities was essential. As an initial attempt, assessment of an EP-reported method was performed. The method defined in the pharmacopoeia was not successful because the separation between the analytes with respect to resolution, symmetry, and peak purity was not satisfactory. Therefore, specific variations were made to the pharmacopoeia-listed method to achieve resolution between all the impurities and chlorthalidone. The finalised method included the following changes: (1) the mobile phase was premixed at a ratio for mobile phase A (65 : 35) and mobile phase B (50 : 50) and (2) the gradient programme time points were increased for complete and effective separation of the impurities. This developed method was suitable for the impurities present in chlorthalidone API and the chlorthalidone tablet formulation.

### 3.1. Characterisation of In-House Impurities

The stage I, stage II, and stage III intermediate products generated during synthesis were identified, characterised, and confirmed with IR, ^1^H-NMR, and mass spectrometry techniques (spectra obtained are added in Supplementary Materials).

#### 3.1.1. Characteristic Data of IR and ^1^H NMR for the Chlorthalidone API Stage-I In-House Process-Related Impurity

  IR: 3091.89 (aromatic C-H stretch), 1720.50 (C=O amide stretch), and 688.59 (para-disubstituted aromatic C-H bend).
 
^1^H-NMR: 7.515–7.494 (t, aromatic 1H), 7.695 (d, aromatic 4H), 8.048–8.025 (d, aromatic 2H), and 8.371–8.349 (d, aromatic 1H).

#### 3.1.2. Characteristic Data of IR and ^1^H NMR for the Chlorthalidone API Stage-II In-House Process-Related Impurity

   IR: 3078.9 (secondary amine N-H stretch), 1678.72 (C=O stretch for amide), 2983.58 (C-H stretch for aromatics), and 750.59 (para-disubstituted aromatic C-H bend).
 
^1^H-NMR: 5.764 (s, amine 1H), 9.07 (s, aromatic 1H), 7.70–7.72 (d, aromatic 2H), 7.55–7.569 (d, aromatic 2H), 7.528–7.428 (d, aromatic 2H), and 7.288–7.489 (d, aromatic 2H).

#### 3.1.3. Characteristic Data of IR and ^1^H NMR for the Chlorthalidone API Stage-III In-House Process-Related Impurity

  IR: 3369.64, 3342.64 (primary amine N-H stretch), 3170.97 (secondary amine N-H stretch), 1674.21 (C=O stretch for amide), 3070.68 (C-H stretch for aromatics), 1521.84, 1614.42 (C-C multiple bond stretch for aromatics), and 732.95 (para-disubstituted C-H bend aromatics).
 
^1^H-NMR: 9.15 (s, 2H, SO_2_NH_2_), 5.89 (d, 1H, amine H at 8), and 7.307–7.84 (aromatic H).

### 3.2. Method Validation

Since the proposed method was a modified EP method, the EP-listed impurities were determined based on their RRTs, and the method was only validated for in-house process-related impurities. The validation study was carried out based on the given ICH guideline Q2R1.

#### 3.2.1. Specificity

**Figure 3:**
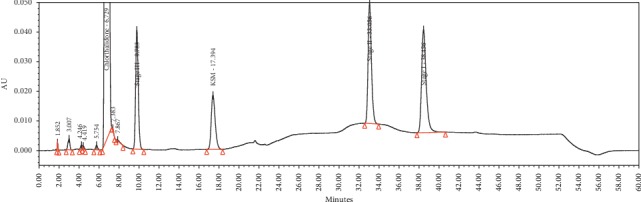
Spiked solution containing the chlorthalidone API and process-related impurities.

**Figure 4:**
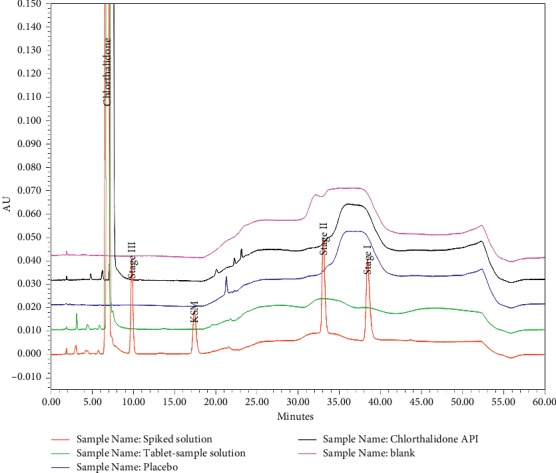
Overlay chromatograms of the blank solution, chlorthalidone tablet placebo solution, chlorthalidone tablet formulation solution, and the chlorthalidone API.

Specificity is the ability to unequivocally assess the analyte in the presence of components that are expected to be present. The impurities and chlorthalidone working standard solutions were injected to determine the retention time (RT), relative retention time (RRT), and resolution, as is shown in Figure 3. The purity angle was within the purity threshold limit obtained for the impurities; this confirmed the analyte peak homogeneity. The EP-specified impurities were monitored based on their reported RRTs. The chromatograms of the chlorthalidone API and chlorthalidone tablet solution showed absence of the EP-specified impurities. The peak results for the spiked solution containing the chlorthalidone API and in-house process-related impurities are given in Table 3. The overlay chromatograms of the blank solution, chlorthalidone tablet placebo solution, chlorthalidone tablet formulation solution, and the chlorthalidone API are given in Figure 4.

#### 3.2.2. Force Degradation Results

Forced degradation studies were performed on chlorthalidone to identify the major degradants and to provide an indication of the stability of the proposed method [21]. The stress conditions employed for the degradation study included acid hydrolysis (1.0 N HCl), base hydrolysis (1.0 N NaOH), and oxidative hydrolysis (30% v/v H_2_O_2_). The main objective was to achieve ∼10% degradation.

**Figure 5:**
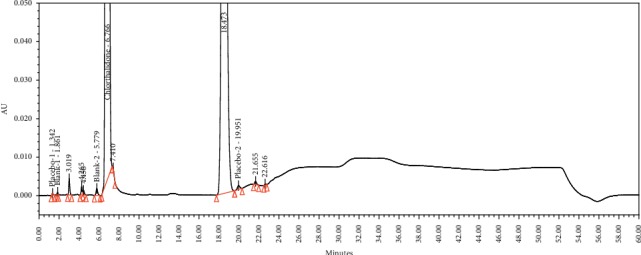
Chromatogram of the acid-stressed chlorthalidone API sample.

The degradation study was carried out on the API and formulation with a concentration of 1,000 *μ*g/mL. The known and unknown impurities in the API and formulation were determined before initiation of the degradation studies. There was no significant degradation observed due to alkali or oxidative hydrolysis or thermal or photolytic conditions. However, the API and tablets were susceptible to degradation under acid hydrolysis conditions, as is shown in Figure 5.

There was an unknown degradation peak observed at an RT of 18.473 minutes that showed 9.56% degradation in 2 mL 1 N HCl after the 30 minute benchtop stress condition.

This unknown degradation peak did not interfere with the in-house process-related impurities. Additionally, there were no peaks or interferences observed when the placebo or blank was injected; thus, the method was proven to be specific.

#### 3.2.3. Linearity

Calibration curves were drawn for chlorthalidone and the two in-house process-related impurities. The range of chlorthalidone was 1–2.8 *μ*g/mL, and both in-house impurities ranged from 0.75 to 2.1 *μ*g/mL. The calculated correlation coefficient was greater than 0.999 for both impurities. The *R*^2^ values for chlorthalidone and the stage II and III impurities were 0.9978, 0.9985, and 0.9986, respectively. The obtained results indicated that there was an excellent linear relationship between the peak area and concentrations for all the components.

#### 3.2.4. Precision

The precision of an analytical procedure expresses the agreement between a series of measurements obtained from multiple samplings from the same homogeneous sample under prescribed conditions. For system precision, the %RSD for peak area of the chlorthalidone standard was 0.098%. There was no significant difference between the mean and individual values, and thus, the method was suitably precise. In the case of method precision, the %RSD for the %w/w impurities present was 1.02% and 0.8200%, which was less than 2%, and thus complied with the acceptance criteria, as is depicted in Table 4.

#### 3.2.5. Accuracy

Accuracy was determined by carrying out recovery studies. The accuracy was evaluated at three levels including approximately 0.5%, 1.0%, and 1.5%, which reflect approximately 50%, 100%, and 150%, respectively, compared to a specification limit of 1.0%. At each recovery level, the % recovery of both impurities was NLT 97.0% and NMT 102.0% for the chlorthalidone API. Similarly, the chlorthalidone tablet formulation % recovery for both impurities was found to be NLT 80.0% and NMT 120.0%. The results suggest that the method is accurate and can quantify impurities in both chlorthalidone API and formulation. The % recovery of the impurities in the chlorthalidone API and tablet is summarised in Tables 5 and 6, respectively.

#### 3.2.6. LOQ and LOD

The quantitation limit is the lowest amount of analyte in a sample that can be quantitatively determined with suitable precision and accuracy. The detection limit is the lowest amount of analyte in a sample that can be detected but not necessarily quantitated as an exact value. The LOQ of chlorthalidone was observed as 0.4 *μ*g/mL and 0.32 *μ*g/mL, of the stage II impurity as 0.3 *μ*g/mL and 0.24 *μ*g/mL, and of the stage III impurity as 0.3 *μ*g/mL and 0.25 *μ*g/mL by the visual and calibration curve methods, respectively. The LOD of chlorthalidone was observed as 0.12 *μ*g/mL and 0.10 *μ*g/mL, of the stage II impurity as 0.09 *μ*g/mL and 0.085 *μ*g/mL, and of the stage III impurity as 0.09 *μ*g/mL and 0.083 *μ*g/mL by the visual and calibration curve methods, respectively.

#### 3.2.7. Robustness

The robustness is a measure of whether an analytical method is unaffected by small but deliberate variations in method parameters and indicates the reliability of the method for routine analysis of samples. The robustness results summarised in Table 7 indicate that there were no significant differences between the method precision and robustness results. Thus, the method is suitably precise and reproducible even after slight variations to the buffer pH, flow rate, and wavelength.

#### 3.2.8. Solution Stability

The % difference in chlorthalidone, stage II process impurity, and stage III process impurity was 1.0%, 5.88%, and 4.17%, respectively, which were within the 10% acceptance criteria. The stability studies showed that the chlorthalidone standard solution was stable up to 40 hrs and that the impurity-spiked solutions were stable up to 36 hrs at 25°C.

#### 3.2.9. Filter Selection Study

Filter compatibility studies were performed with 0.45 *μ*m nylon filters and 0.45 *μ*m PVDF filters using chlorthalidone standard solution and in-house process impurity working standard solutions. The similarity factor results for the chlorthalidone standard solution were 1.00, which was within 0.95 to 1.05 acceptance criteria. The calculated absolute % difference between the unfiltered and filtered solutions was within the specification limits. Hence, both 0.45 *μ*m nylon filter and 0.45 *μ*m PVDF filter were compatible and could be used for routine analysis.

#### 3.2.10. Stability Studies

The stability studies provide evidence of the quality of an API or a finished pharmaceutical product, which may differ with time due to a variety of environmental factors such as temperature, humidity, and light. Thus, long-term and accelerated stability studies were performed on the API and formulation. The stability studies, as specified in Table 8, revealed that the % impurity levels are within the EP and in-house specification limits for both API and formulation.

## 4. Conclusion

The available literature did not report a single method for the determination of chlorthalidone impurities and degradants. A newly modified RP-HPLC method could separate all the analytes, i.e., pharmacopoeia-listed, in-house-related, and degradation impurities, with acceptable resolution and tailing factor. The degradation study revealed that the formed unknown impurity can be well resolved with the same method without any interference from the in-house process-related impurities. The proposed method is precise and accurate for detecting possible known and unknown impurities in both chlorthalidone API and tablet. Thus, this developed method can be used for routine impurity analysis with the chlorthalidone API and tablet formulation.

## Figures and Tables

**Table 1:** Impurities.

Name	Structure	IUPAC	Specification limits

Impurities listed in European pharmacopoeia			
Impurity B	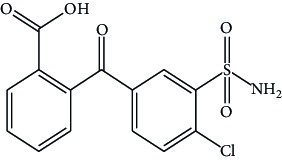	2-(4-Chloro-3-sulfamoylbenzoyl) benzoic acid.	Not more than 0.7%
Impurity G	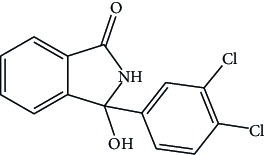	(3RS)-3-(3-Dichlorophenyl)-3-hydroxy-2,3-dihydro-1H-isoindol-1-one	Not more than 0.2%
Impurity J	It is a specified but unidentified impurity that elutes at RRT 0.9 with reference to chlorthalidone		Not more than 0.3%
In-house process-related impurities			
Intermediate stage II	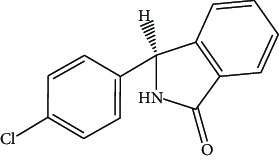	3-(4-Chlorophenyl) isoindolin-1-one	Not more than 0.15%
Intermediate stage III	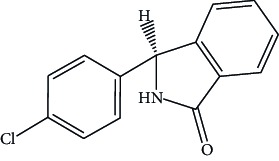	3-(4-Chlorophenyl)-5-sulfonamide isoindoline -1-one	Not more than 0.15%

**Table 2:** Mobile phase gradient elution programme.

Gradient Programme: for blank solution, placebo solution, and sample solution		
0.01	100	0
16.0	100	0
21.0	0	100
50.0	0	100
52.0	100	0
60.0	100	0

**Table 3:** Peak results for the spiked solution containing the chlorthalidone API and in-house process-related impurities.

Sample name	Area	RT	RRT	Resolution	Theoretical plates	Tailing factor	Purity angle	Purity threshold
Chlorthalidone	55150545	6.729	—	2.54	4240	1.21	9.786	1.001
Stage II	938956	33.056	4.912	23.10	47507	1.26	0.183	1.129
Stage III	727129	9.783	1.453	3.20	6770	1.17	0.180	1.108

**Table 4:** Precision results.

Sr. no.	System precision	Method precision	
	area	Stage II process impurity (%w/w)	Stage III process impurity (%w/w)
	chlorthalidone		
1	143734	0.173	0.250
2	144067	0.168	0.251
3	144083	0.172	0.253
4	143872	0.169	0.255
5	143988	0.170	0.258
6	144079	0.171	0.256
Mean	143970.5	0.1705	0.254
SD	141.19	0.001871	0.00208
%RSD	0.098	1.02	0.8200

**Table 5:** Results for the % recovery with the chlorthalidone API.

	% recovery								
	50%			100%			150%		
Stage II process impurity	99.90	100.08	100.27	99.47	99.77	99.83	97.74	98.62	99.49
Mean % recovery	100.08			99.69			98.62		
SD	988.55			2102.35			14152.62		
%RSD	0.183			0.195			0.886		
Stage III process impurity	99.47	99.75	100.14	100.75	100.47	100.99	98.85	99.91	99.47
Mean % recovery	99.78			100.74			99.42		
SD	1892.40			2816.47			8592.28		
%RSD	0.3516			0.2591			0.5340		

**Table 6:** Results for the % recovery with the chlorthalidone tablets.

	% recovery								
	50%			100%			150%		
Stage II process impurity	100.03	98.39	98.71	99.29	99.34	99.44	99.49	100.47	100.71
Mean % recovery	99.05			99.36			100.22		
SD	4689.33			815.064			10458.61		
%RSD	0.8776			0.0760			0.6447		
Stage III process impurity	98.91	98.89	98.60	99.45	99.63	99.91	98.80	99.22	99.66
Mean % recovery	98.25			99.62			99.23		
SD	1035.37			1819.20			6980.63		
%RSD	0.194			0.169			0.434		

**Table 7:** Robustness values for the chlorthalidone and the in-house process-related impurities.

Sr. no.	Name	Control	pH		Flow rate		Wavelength	
		RRT						
			pH 5.3	pH 5.7	1.2 mL/min	1.6 mL/min	218 nm	222 nm
1	Chlorthalidone	1.0	1.0	1.0	1.0	1.0	1.0	1.0
2	Stage II process impurity	4.91	4.98	4.89	4.90	4.93	4.92	4.91
3	Stage III process impurity	1.45	1.49	1.42	1.45	1.44	1.46	1.45

**Table 8:** Stability study results.

Impurities	40°C/75%RH					25°C/60%RH		Limit
	Initial	1 M	2 M	3 M	6 M	3 M	6 M	
*For API*								
Impurity B (%w/w)	ND	ND	ND	ND	ND	0.01	0.01	NMT 0.10%
Stage II (%w/w)	ND	ND	ND	0.0	0.0	ND	ND	NMT 0.15%
Stage III (%w/w)	ND	ND	0.01	0.01	0.01	ND	ND	NMT 0.15%
Single large unknown impurity (%w/w)	0.07	0.05	0.03	0.05	0.06	0.05	0.04	NMT 0.10%
Total impurities (%w/w)	0.29	0.20	0.21	0.26	0.27	0.17	0.15	NMT 0.5%
*For formulation*								
Impurity B (%w/w)	ND	ND	ND	ND	ND	0.01	0.01	NMT 0.10%
Stage II (%w/w)	ND	ND	ND	0.0	0.0	ND	ND	NMT 0.15%
Stage III (%w/w)	0.01	0.01	0.01	0.02	0.03	ND	ND	NMT 0.15%
Single large unknown impurity (%w/w)	0.05	0.05	0.07	0.06	0.09	0.06	0.05	NMT 0.10%
Total impurities (%w/w)	0.30	0.28	0.27	0.26	0.25	0.27	0.25	NMT 0.5%

ND: not detected.

## Data Availability

The authors confirm that the data supporting the findings of this study are available within the article and/or its supplementary information files.

## Supplementary Materials

Supplementary Materials Figure 3.1: Characterisation of the chlorthalidone API and in-house process-related impurity. Figure 3.1.1: IR spectra of the chlorthalidone API stage-I in-house process-related impurity. Figure 3.1.2: 1H NMR spectra of the chlorthalidone API stage-I in-house process-related impurity. Figure 3.1.3: Mass spectra of the chlorthalidone API stage-I in-house process-related impurity. Figure 3.1.4: Characterisation of the chlorthalidone API stage-II in-house process-related impurity. Figure 3.1.5: IR spectra of the chlorthalidone API stage-II in-house process-related impurity. Figure 3.1.6: 1H NMR spectra of the chlorthalidone API stage-II in-house process-related impurity. Figure 3.1.7: Mass spectra of the chlorthalidone API stage-II in-house process-related impurity. Table 3.1.1: Interpretation of the IR and 1H NMR data for the chlorthalidone API stage-II in-house process-related impurity. Figure 3.1.8: Characterisation of the chlorthalidone API stage-III in-house process-related impurity. Figure 3.1.9: IR spectra of the chlorthalidone API stage-III in-house process-related impurity. Figure 3.1.10: 1H NMR spectra of the chlorthalidone API stage-III in-house process-related impurity. Figure 3.1.11: Mass spectra of the chlorthalidone API stage-III in-house process-related impurity. Table 3.1.2: Interpretation of the IR and 1H NMR data for the chlorthalidone API stage-III in-house process-related impurity.
