# Supplementary material for: A Validated RP-HPLC Stability Method for the Estimation of Chlorthalidone and Its Process-Related Impurities in an API and Tablet Formulation
Source: Int J Anal Chem. 2020 Apr 10;2020:3593805. doi: 10.1155/2020/3593805 (PMC7171635; doi:10.1155/2020/3593805)
Supplement: Supplementary Materials — Figure 3.1: Characterisation of the chlorthalidone API and in-house process-related impurity. Figure 3.1.1: IR spectra of the chlorthalidone API stage-I in-house process-related impurity. Figure 3.1.2: 1H NMR spectra of the chlorthalidone API stage-I in-house process-related impurity. Figure 3.1.3: Mass spectra of the chlorthalidone API stage-I in-house process-related impurity. Figure 3.1.4: Characterisation of the chlorthalidone API stage-II in-house process-related impurity. Figure 3.1.5: IR spectra of the chlorthalidone API stage-II in-house process-related impurity. Figure 3.1.6: 1H NMR spectra of the chlorthalidone API stage-II in-house process-related impurity. Figure 3.1.7: Mass spectra of the chlorthalidone API stage-II in-house process-related impurity. Table 3.1.1: Interpretation of the IR and 1H NMR data for the chlorthalidone API stage-II in-house process-related impurity. Figure 3.1.8: Characterisation of the chlorthalidone API stage-III in-house process-related impurity. Figure 3.1.9: IR spectra of the chlorthalidone API stage-III in-house process-related impurity. Figure 3.1.10: 1H NMR spectra of the chlorthalidone API stage-III in-house process-related impurity. Figure 3.1.11: Mass spectra of the chlorthalidone API stage-III in-house process-related impurity. Table 3.1.2: Interpretation of the IR and 1H NMR data for the chlorthalidone API stage-III in-house process-related impurity. [file 3593805.f1.pdf]

Figure 3.1: Characterisation of the Chlorthalidone API & In-house process-related impurity.

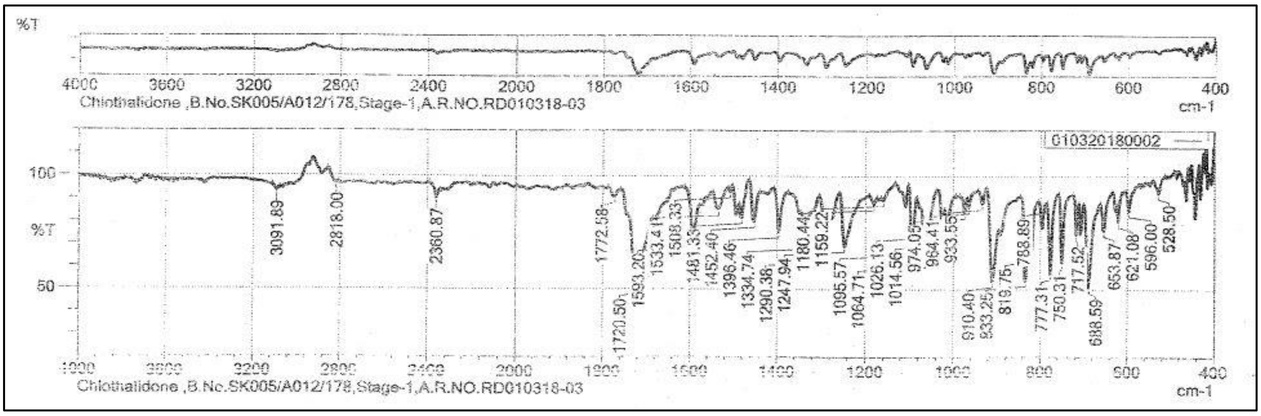

Figure 3.1.1 IR spectra of the Chlorthalidone API Stage- I In-house process-related impurity.

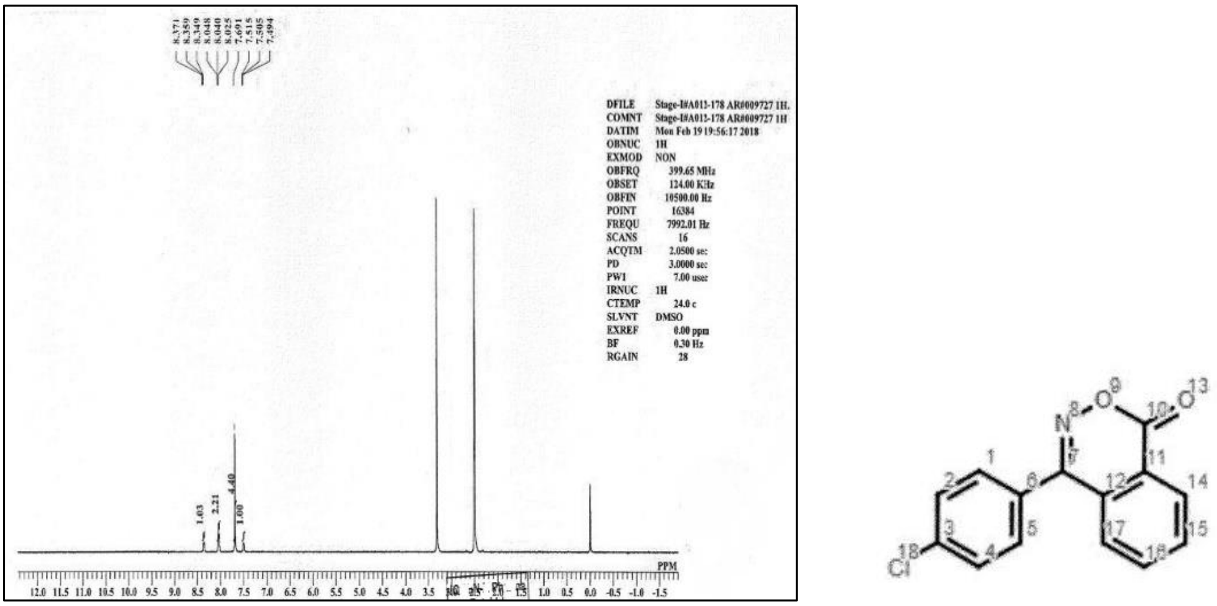

Figure 3.1.2: ^{1}H NMR spectra of the Chlorthalidone API Stage-I In-house process-related impurity.

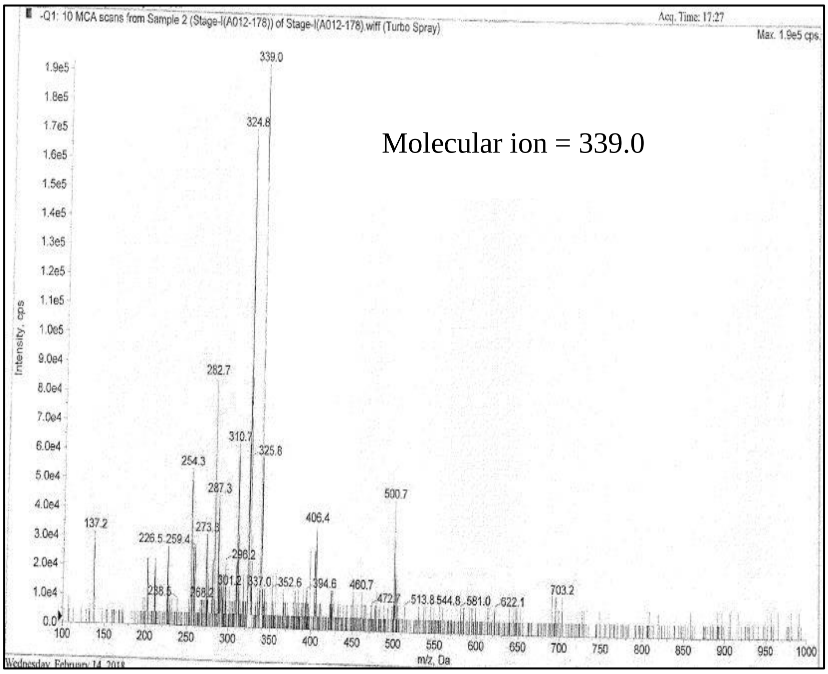

Figure 3.1.3: Mass spectra of the Chlorthalidone API Stage-I In-house process-related impurity.

Figure 3.1.4: Characterisation of the Chlorthalidone API Stage-II In-house process-related impurity.

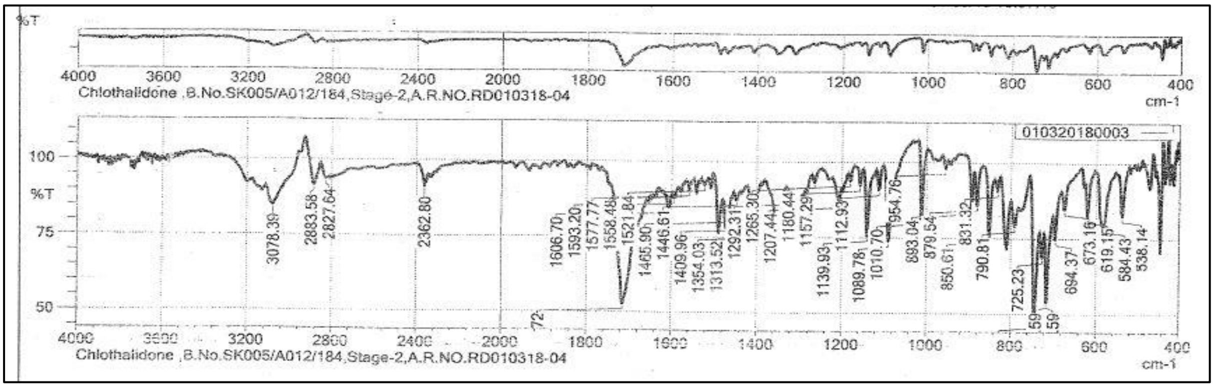

Figure 3.1.5: **IR spectra of the Chlorthalidone API Stage-II In-house process-related impurity.**

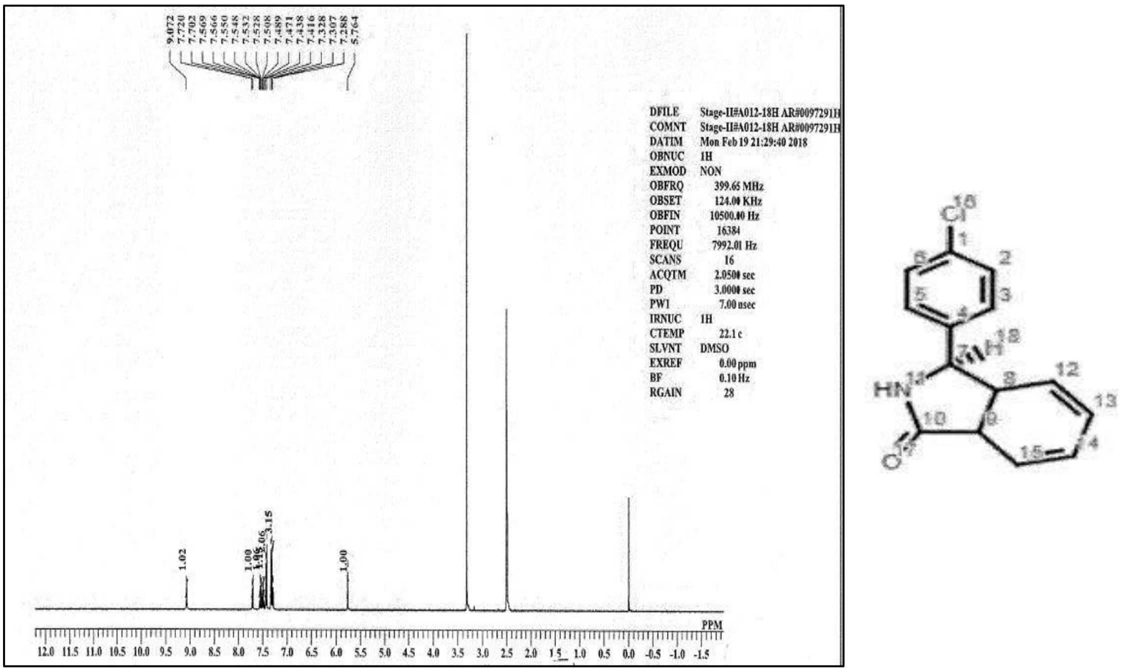

Figure 3.1.6: ^{1}H NMR spectra of the Chlorthalidone API Stage-II In-house process-related impurity.

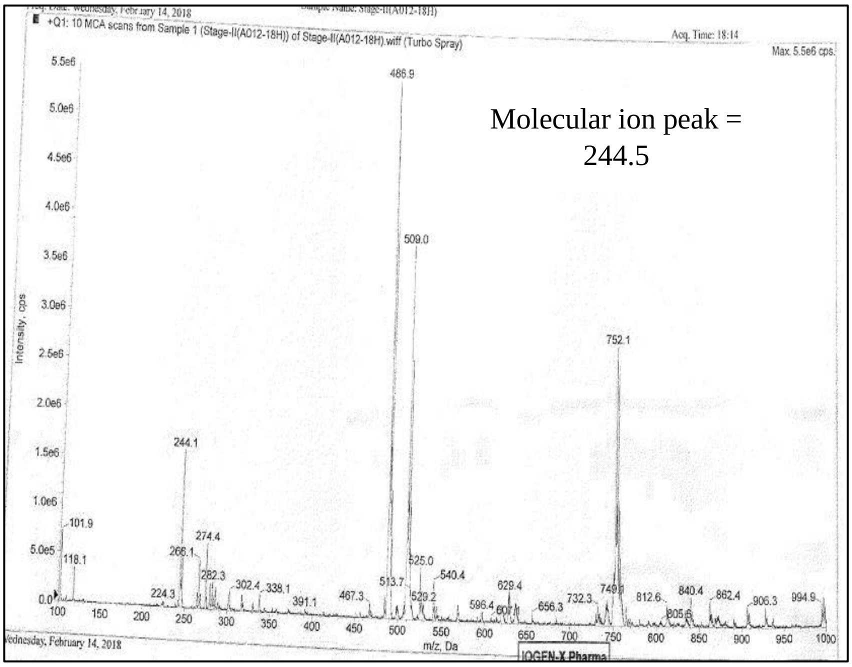

Figure 3.1.7: Mass spectra of the Chlorthalidone API Stage-II In-house process-related impurity.

Table 3.1.1: Interpretation of the IR and ^{1}H NMR data for the Chlorthalidone API Stage-II In-house process-related impurity.

Stage II							
IR data			NMR data				
Peak No.	V (cm^{-1})	Functional group	Labelled proton	No. of protons	δ (ppm)	M	Functional group
1	3078.39	Secondary amine N-H stretch	11	1	5.764	s	Amine H at 11
2	1678.72	C=O stretch for amide	8	1	9.07	s	Aromatic H
3	2983.58	C-H stretch for aromatics	2 & 6	2	7.72-7.70	d	Aromatic H
4	750.59	Para-disubstituted C-H bend aromatics	3 &5	2	7.569-7.55	d	Aromatic H
			12 & 15	2	7.548-7.528	d	Aromatic H
			13 & 14	2	7.489-7.288	t	Aromatic H

V= Wavenumber, M= Multiplicity; S = singlet; D= doublet; T= triplet

Figure 3.1.8: Characterisation of the Chlorthalidone API Stage-III In-house process-related impurity.

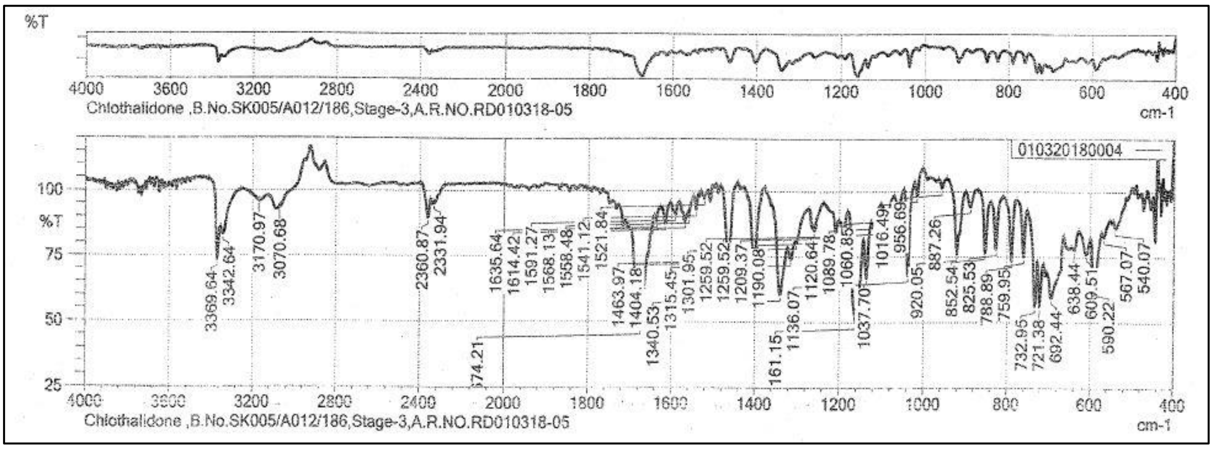

Figure 3.1.9: **IR spectra of the Chlorthalidone API Stage-III In-house process-related impurity.**

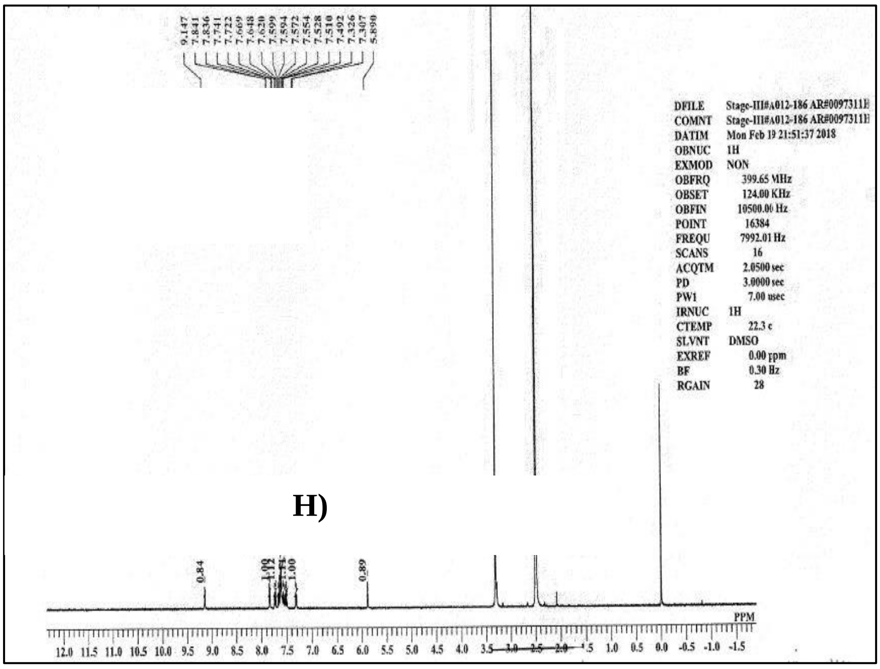

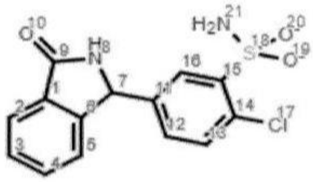

Figure 3.1.10: ^{1}H NMR spectra of the Chlorthalidone API Stage-III In-house process-related impurity.

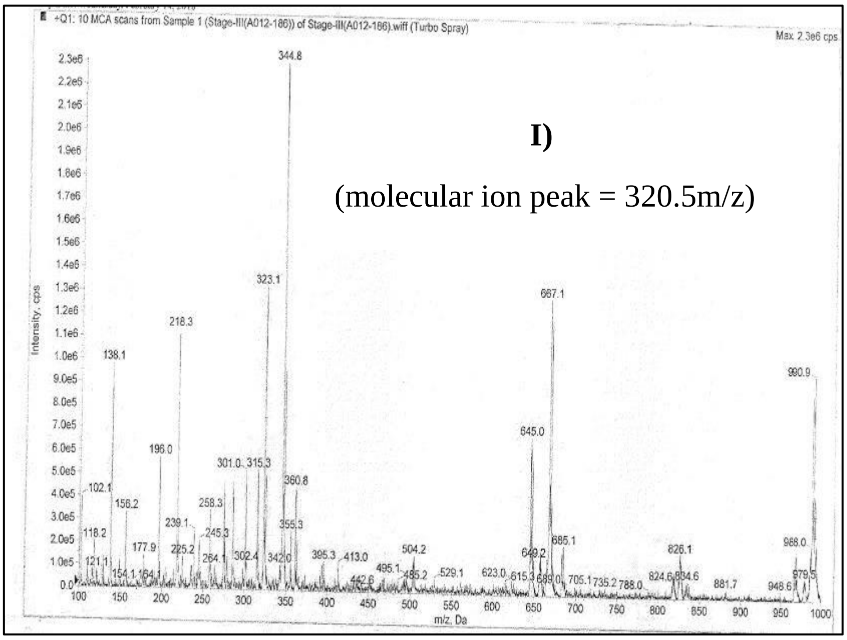

Figure 3.1.11: Mass spectra of the Chlorthalidone API Stage-III In-house process-related impurity.

Table 3.1.2: Interpretation of the IR and ^{1}H NMR data for the Chlorthalidone API Stage-III In-house process-related impurity.

**Stage III**							
**IR data**			**NMR data**				
**Peak No.**	**V (cm^{-1})**	**Functional group**	**Labelled proton**	**No. of protons**	**δ (ppm)**	**M**	**Functional group**
1	3369.64,3342.64	Primary amine N-H stretch	21	2	9.15	s	SO_2NH_2
2	3170.97	Secondary amine N-H stretch	8	1	5.89	d	Amine H at 8
3	1674.21	C=O stretch for amide	7	1	7.307	d	Aromatic H
4	3070.68	C-H stretch for aromatics	3	1	7.492-7.528	t	Aromatic H
5	1521.84,1614.42	C-C multiple bond stretch for aromatics	4	1	7.554	d	Aromatic H
6	732.95	Para-disubstituted	5	1	7.572	d	Aromatic H

		C-H bend aromatics					
			2	1	7.594-7.626	d	Aromatic H
			12	1	7.648-7.669	d	Aromatic H
			13	1	7.74-7.72	d	Aromatic H
			16	1	7.84-7.83	d	Aromatic H

V= Wavenumber, M= Multiplicity; s= singlet; d= doublet; t= triplet
